# Pennsylvania Medical College

**Published:** 1852-05

**Authors:** 


THE MEDICAL EXAMINER.

PHILADELPHIA, MAY, 1852.

### PENNSYLVANIA MEDICAL COLLEGE.

We learn, with much pleasure, that the Faculty of this Institution is about to be strengthened by the accession of Drs. F. G. Smith, J. M. Allen, and J. J. Reese, who have accepted the Professorships of *Institutes of Medicine, Anatomy,* and *Medical Chemistry and Pharmacy.* The two last named chairs have been lately vacated by death and resignation; the chair of Institutes is a new creation. These appointments are in every respect excellent, and cannot fail to advance the interests of the school in question. Drs. Smith and Allen have for many years filled lectureships in the Philadelphia Association for Medical Instruction, on the branches to which they are now translated in the Pennsylvania College; and the reputation which they have established, as eloquent, erudite, and judicious teachers, is second to none in the country. Dr. Reese has been for some time connected with the Philadelphia Medical Institute as a lecturer on Materia Medica and Therapeutics, and has been most favorably distinguished, both as a teacher and as a writer on this and other branches of medicine. His success in the chair of Materia Medica is a guarantee of his ability to do full justice to the kindred subject which he now undertakes.

We may also say, in noticing these appointments, that the high personal character and professional standing of the gentlemen named, cannot but give strength and popularity to any school with which they are connected. The Pennsylvania Medical College has already attained a most respectable position, and under this new organization, may rapidly anticipate a brilliant career. We are sure that it will have the cordial good wishes of the entire profession in Philadelphia. B.

We have the pleasure of laying before our readers, in the present No., a communication from Dr. *E. Brown-Sequard,* of Paris, now on a visit to this city, of which his father was a native.

This gentleman has attained a distinguished place amongst the experimental physiologists of the day, as one entitled to the utmost confidence in his observations and statements. He holds the high position of perpetual Secretary to the "Societé de Biologie" in Paris, and his communications have been extensively copied into all the standard treatises on Physiology, as well as into the foreign and domestic journals. We believe that it is his intention to give instruction in this department of Medical Science, which we are sure, from our knowledge of him, will prove both interesting and useful.

